# Jellyfish Body Plans Provide Allometric Advantages beyond Low Carbon Content

**DOI:** 10.1371/journal.pone.0072683

**Published:** 2013-08-13

**Authors:** Kylie A. Pitt, Carlos M. Duarte, Cathy H. Lucas, Kelly R. Sutherland, Robert H. Condon, Hermes Mianzan, Jennifer E. Purcell, Kelly L. Robinson, Shin-Ichi Uye

**Affiliations:** 1 Australian Rivers Institute-Coasts and Estuaries & Griffith School of Environment, Griffith University, Gold Coast, Queensland, Australia; 2 The UWA Oceans Institute, the University of Western Australia, Perth, Western Australia, Australia; 3 Department of Global Change Research, Instituto Mediterráneo de Estudios Avanzados, Universitat de les Illes Balears, Consejo Superior de Investigaciones Cientificas, Esporles, Spain; 4 Faculty of Marine Sciences, King Abdulaziz University, Jeddah, Saudi Arabia; 5 National Oceanography Centre Southampton, University of Southampton, Southampton, United Kingdom; 6 Oregon Institute of Marine Biology and Institute of Ecology and Evolution, University of Oregon, Eugene, Oregon, United States of America; 7 Dauphin Island Sea Laboratory, Dauphin Island, Alabama, United States of America; 8 Instituto Nacional de Investigación y Desarrollo Pesquero, Mar del Plata, Argentina; 9 Shannon Point Marine Center, Western Washington University, Anacortes, Washington, United States of America; 10 Department of Marine Science, University of Southern Mississippi, Stennis, Mississippi, United States of America; 11 Graduate School of Biosphere Science, Hiroshima University, Higashi-hiroshima, Japan; Utah State University, United States of America

## Abstract

Jellyfish form spectacular blooms throughout the world’s oceans. Jellyfish body plans are characterised by high water and low carbon contents which enables them to grow much larger than non-gelatinous animals of equivalent carbon content and to deviate from non-gelatinous pelagic animals when incorporated into allometric relationships. Jellyfish have, however, been argued to conform to allometric relationships when carbon content is used as the metric for comparison. Here we test the hypothesis that differences in allometric relationships for several key functional parameters remain for jellyfish even after their body sizes are scaled to their carbon content. Data on carbon and nitrogen contents, rates of respiration, excretion, growth, longevity and swimming velocity of jellyfish and other pelagic animals were assembled. Allometric relationships between each variable and the equivalent spherical diameters of jellyfish and other pelagic animals were compared before and after sizes of jellyfish were standardised for their carbon content. Before standardisation, the slopes of the allometric relationships for respiration, excretion and growth were the same for jellyfish and other pelagic taxa but the intercepts differed. After standardisation, slopes and intercepts for respiration were similar but excretion rates of jellyfish were 10× slower, and growth rates 2× faster than those of other pelagic animals. Longevity of jellyfish was independent of size. The slope of the allometric relationship of swimming velocity of jellyfish differed from that of other pelagic animals but because they are larger jellyfish operate at Reynolds numbers approximately 10× greater than those of other pelagic animals of comparable carbon content. We conclude that low carbon and high water contents alone do not explain the differences in the intercepts or slopes of the allometric relationships of jellyfish and other pelagic animals and that the evolutionary longevity of jellyfish and their propensity to form blooms is facilitated by their unique body plans.

## Introduction

Jellyfish (cnidarian medusae and ctenophores) have been forming blooms in the world’s oceans for 500 million years and they frequently represent a large proportion of the pelagic consumer biomass. The major feature that distinguishes jellyfish from most other pelagic metazoans is the very high water (>95%) and low carbon (usually <1% of wet weight) content of their bodies [1]. By incorporating large volumes of water into their bodies, jellyfish grow to sizes that are disproportionately larger than other animals relative to their carbon content (termed ‘faking giants’ [2]). Large body size confers many ecological advantages including being less vulnerable to predators, the ability to capture more or larger prey and, specific to the pelagic environment, the potential to operate at higher Reynolds numbers (Re). Thus, using water to increase their body size potentially offers many adaptive advantages to jellyfish and may contribute to their evolutionary longevity, widespread distribution and abundance in the world’s oceans [2,3].

Most physiological and ecological processes scale allometrically with body size [4]. Allometric relationships can predict a broad range of functional properties of animals including rates of respiration and growth, efficiency of locomotion, and even duration of sleep, pointing to body size as a key trait with great functional consequences [4]. Most allometric relationships are scaled to organisms’ mass, whether represented by wet, dry, or carbon mass. The choice of mass unit for comparison has minimal effect when comparing organisms with similar water and carbon contents but is critical when comparisons include animals, such as jellyfishes, that have vastly different carbon and water contents. The high water content of jellyfish has thus caused them to differ from other metazoans when they are incorporated into general allometric relationships based on wet or dry mass and the degree of difference varies with the unit chosen. Differences in the size-dependence of some functional responses of jellyfish relative to those of other metazoans, however, disappear once their carbon mass, rather than wet or dry mass is used to characterize their body size [2,5]. Comparing how allometric relationships vary when different units of size are used can provide information about how organisms with different body plans undertake key ecological and functional processes. For example, jellyfish have slower respiration and instantaneous clearance rates than fish when scaled to wet mass but rates are similar once scaled to carbon mass indicating that, although jellyfish have less efficient prey capture mechanisms than fish, enlarging their bodies with water enables jellyfish to maintain high prey contact rates [2]. Some studies have suggested that carbon should be used as the universal metric of size when including jellyfish in physiological studies [2,6]. Due to the very dilute carbon present in gelatinous organisms, however, scaling to carbon will under-represent the physical size of jellyfish relative to other animals and, therefore, may hinder consideration of many of the physiological and ecological functions and benefits associated with large body size which do not directly depend on carbon.

The physiological attributes of jellyfish body plans extend beyond their high water and low carbon content. For example, many jellyfish exhibit instantaneous growth rates exceeding 0.3 d^-1^ [7]. This growth rate exceeds that of many smaller zooplankton despite jellyfish having carbon contents that are orders of magnitude greater than those of smaller zooplankton. Cnidarian medusae may also deviate greatly from the general scaling between organismal size and life span because even very large medusae generally live for less than one year (e.g. [8]). Moreover, the unique modes of locomotion of jellyfish (‘jet propulsion’ in medusae and ciliary movements in ctenophores) suggest that the allometry of swimming will differ between jellyfish and other pelagic taxa. Overall, these likely allometric differences suggest that the jellyfish body plan may have effects on key ecological traits that cannot be fully explained by simply correcting for low carbon content.

Here we compare the allometric relationships of jellyfish and other pelagic animals for a range of key functional properties of planktonic animals including rates of respiration, excretion, growth, longevity, swimming velocity and Reynolds numbers and test whether the relationships of jellyfish deviate in slope (i.e. allometry) and/or intercept. We then test whether these deviations disappear once jellyfish size is scaled to its carbon mass. We predict that metabolic processes, such as respiration and excretion, will be tightly coupled to carbon content (i.e. both slopes and intercepts would be similar when scaled to carbon mass). Because jellyfish maintain similar rates of respiration and clearance to other zooplankton when scaled to carbon mass [2] it is also anticipated that the slope and intercept for growth will also be similar to that of other pelagic taxa when scaled to carbon mass. However, longevity and average swimming velocity are predicted to scale differently (i.e. both slope and intercepts would differ) regardless of the unit of size used for comparison due to fundamental differences in modes of locomotion between jellyfish and other taxa and due to the observation that most populations of jellyfish display distinct seasonal cycles in abundance. Moreover, we predict that enlarging their bodies with water may enable jellyfish to operate at higher Reynolds numbers than if their body size was comparable to other animals of similar carbon content. We conclude that jellyfish exhibit allometric differences, of fundamental ecological consequence, that cannot be explained solely by their low carbon content and that these allometric differences may provide insights into the evolutionary and competitive advantages conferred by their unique body plans and may help to explain their propensity to form blooms.

## Materials and Methods

Data on carbon and nitrogen content, respiration, excretion, maximum specific growth rates, longevity, and average cruising velocities of cnidarian medusae (mainly hydrozoans and scyphozoans), ctenophores and pelagic representatives of the phyla Annelida, Arthropoda, Mollusca, Chaetognatha, and Chordata were assembled from the global literature. Although the study aimed to encompass all pelagic gelatinous organisms, data on gelatinous molluscs (pteropods), echinoderms (holothurians) and annelids were too scarce to allow a robust analysis. Moreover, data on pelagic thaliaceans were also excluded due to inconsistencies in some reported biometric conversions for that group. For the full dataset see Dataset S1 and Appendix S1 in Supporting Information. With the exception of growth rates, larvae and juveniles were excluded from analyses because mass-specific respiration rates of early life history stages are usually higher due to their rapid growth [9] and ontogenetic changes in allometry could confound interspecific comparisons. Metabolic rates of medusae are consistent over large depth ranges [10] but metabolic rates of many visually- orientating pelagic animals decrease 1-2 orders of magnitude with depth [11,12] (Figure S1; Dataset S2). Consequently only epipelagic representatives of taxa other than jellyfish were included in the analyses. When multiple measurements for a single taxon were available from a study, data for the largest individuals were used. This was done to avoid over-representation of well-studied species within the datasets. The number of data points for jellyfish in each analysis varied from 18 (for excretion rates) to 48 (for respiration rates). With the exception of swimming velocities and longevity, jellyfish containing symbiotic dinoflagellates (zooxanthellae) were omitted from analyses because symbionts influence the metabolic functions of their hosts [13].

To compare the allometric scaling across disparate taxa, animal sizes were standardized to their equivalent spherical diameter (ESD), which was defined as the diameter of a sphere with a volume equivalent to that of the animal. Although allometric relationships are more commonly presented using metrics of mass (e.g. wet weight, dry weight, carbon mass), mass was not a suitable metric to use in the current study because the high water and low carbon content of jellyfish would result in different allometric relationships for jellyfish, depending on which metric was selected for use. In turn, this would confound attempts to compare allometric relationships between jellyfish and other pelagic taxa. ESD was selected as the metric for comparison, therefore, to provide an independent metric for allometric comparisons between jellyfish and other pelagic taxa.

Animal sizes were usually reported as wet weight (WW), dry weight (DW), carbon content (CC), diameter or length. Due to the exceptionally high water content of jellyfish, WW was assumed to be directly proportional to volume, thereby assuming a density similar to seawater. For jellyfish whose sizes were reported as DW, CC or diameter, algorithms and biometric ratios for conversion of sizes to WW or volumes were derived from [1]. If ratios of WW:DW or CC:DW were required but not available for individual taxa, then averages for the genus or family were applied. Other pelagic animals whose sizes were reported as DW were initially converted to carbon weights and then to volumes by assuming 0.45 g C g DW^-1^ and 0.12 g C cm^-3^ [14]. Squid were often reported in terms of WW and were initially converted to DW (using DW:WW ratios for individual species or the average across species (14.79% [15]) and then to C and volume. Animals that were reported only as lengths were converted to volumes by assuming they formed the shape of a prolate sphere and had aspect ratios of 0.4 for copepods and fish and 0.2 for euphausiids, (as in [16]), and squid. Lengths of squid were reported as dorsal mantle length (DML) and were converted to total length (TL) assuming DML=0.6TL. Details of how individual data were converted are available in Dataset S1.

Temperatures used in the various studies ranged from -1.8 to 30°C. Data for respiration, excretion, growth and longevity were adjusted to account for the different temperatures at which measurements were made using the temperature correction of [17]. If measurements were made over a range of temperatures then the mid-point of the range was used for calculations. Because the range of temperatures over which longevity occurred was rarely reported, longevity data were corrected for temperature by using the average summer sea surface temperature (January for the southern hemisphere and July for the northern hemisphere) for the location of interest using the National Ocean Data Center Las 7.3 global temperature database (http://data.nodc.noaa.gov/las/getUI. do). Estimates of swimming velocity could not be corrected for temperature because the temperatures at which measurements were made were reported for only 30% of studies (Dataset S1). Although temperatures might have been estimated based on the location of the studies, the time of year that measurements were made was often not reported or swimming velocities were undertaken in laboratories, and so estimating temperature would have introduced substantial error.

Maximum weight-specific growth rate was defined as the maximum growth rate reported within any one study. The ESD used in growth analyses was the geometric mean ESD during the period for which growth was derived (*sensu* [7]). Data on growth were compiled from field and laboratory studies. Laboratory studies were only included if animals were fed to excess and field studies where animals were reported to be food-limited were excluded. Measures of longevity were derived from field-based studies that tracked the occurrence of individual cohorts or from studies where individuals could be aged using, for example, otoliths or statoliths. Ctenophores were not included in the analysis of longevity because populations are restocked annually by a subset of individuals that survive over winter. Consequently at least some ctenophores within a population survive for more than one year and this precludes cohort analysis as an effective tool for measuring longevity in ctenophores. Measures of longevity derived from laboratory studies were also excluded because physiological longevity measured in the laboratory is usually much longer than ecological longevity measured in the field [18]. If sizes of the oldest cohort were not reported, the maximum size obtained by that species was used. The relative effects of inertial and viscous forces operating on medusae were evaluated using Reynolds number, Re=LU/ν where L, the characteristic length scale is the ESD, U is mean swimming velocity and ν is the kinematic viscosity of the water (1.05×10^-6^ m s^-1^ for seawater at 20° C *sensu* [19]).

### Statistical analyses

Allometric relationships of jellyfish were compared statistically with those of other pelagic taxa. All data were linearised using log_10_ transformations and analyses of variance (ANOVA) of regressions were used to test for linear relationships separately for jellyfish and other pelagic taxa. The slopes of the allometric relationships were compared using ANOVA to test for the interaction between the dependent variables (jellyfish vs other pelagic animals) and the covariate (ESD) and, if slopes were homogeneous, analyses of covariance (ANCOVA) were used to test for differences in intercepts. If intercepts differed, ESDs for jellyfish were then converted to the ESDs the jellyfish would have if they had a carbon content equivalent to other pelagic taxa. Conversions were made using the relationships between ESD and carbon for jellyfish and other pelagic taxa (Table 1). Intercepts of carbon-adjusted jellyfish and other pelagic animals were then again compared using ANCOVA. Akaike’s Information Criterion (AIC) was then used to compare models that shared a common slope and intercept, common slope and different intercept and different slope and intercept.

**Table 1:** Summary of analyses of variance of regressions for jellyfish (J; raw data), jellyfish standardised for carbon content (SJ), and other pelagic animals (OPA).

Variable	Group	Log a	b (±SEM)	R^2^	ANOVA of regression (P)	Groups compared	Equality of slopes (P)	ANCOVA (P)	AIC (Same slope & intercept)	AIC (Same slope, sep. Intercept)	AIC (Sep. slope, sep. Intercept)
Carbon content	J	-0.03	0.32 ± 0.06	0.93	<0.001	J vs OPA	0.484	<0.001	39.628	-78.69*	-76.69
(mg ind^-1^)	OPA	-0.60	0.33 ± 0.0	1	<0.001						
Nitrogen content	J	0.16	0.32 ± 0.02	0.88	<0.001	J vs OPA	0.627	<0.001	42.16	-30.30	-28.3*0
(mg ind^-1^)	OPA	-0.38	0.33 ± 0.01	0.97	<0.001						
Respiration	J	9.27	2.57 ± 0.13	0.90	<0.001	J vs OPA	0.247	<0.001	234.54	71.36*	73.30
(ml O_2_ ind^-1^ h^-1^)	SJ	10.69	2.47 ± 0.12	0.89	<0.001	SJ vs OPA	0.046	NA	64.86*	66.86	67.29
	OPA	10.72	2.72 ± 0.05	0.98	<0.001						
Excretion	J	9.04	2.60 ± 0.53	0.58	<0.001	J vs OPA	0.923	<0.001	102.96	79.95*	81.95
(μmol NH_4_^+^ ind^-1^ h^-1^)	SJ	10.47	2.50 ± 0.51	0.58	<0.001	SJ vs OPA	0.784	0.001	84.28	78.22*	80.22
	OPA	11.45	2.66 ± 0.19	0.95	<0.001						
Max specific growth	J	10.58	-0.35 ± 0.12	0.25	0.007	J vs OPA	0.452	<0.001	44.89	31.39*	33.39
(d^-1^)	SJ	10.38	-0.34 ± 0.11	0.29	0.007	SJ vs OPA	0.389	0.009	32.42	30.31*	32.31
	OPA	10.03	-0.47 ± 0.09	0.62	<0.001						
Longevity (d)	J	13.47	-0.25 ± 0.12	0.18	0.046	NA					
	OPA	14.14	0.15 ± 0.14	0.04	0.295	NA					
Swimming velocity	J	0.18	0.30 ± 0.13	0.19	0.026	J vs OPA	<0.001	NA	98.0	81.86	68.44*
(cm s^-1^)	OPA	0.65	0.90 ± 0.05	0.91	<0.001						
Reynolds number	J	2.15	1.29 ± 0.12	0.83	<0.001	J vs OPA	<0.001	NA	96.73	78.94	64.09*
	SJ	1.77	1.32 ± 0.12	0.84	<0.001	J vs SJ	0.872	0.002			
	OPA	2.63	1.89 ± 0.05	0.98	<0.001						

NA = analysis not applicable. Relationships for carbon and nitrogen content take the form of Log ESD (cm) = log a + b × log (C or N) (g ind^-1^). Relationships for other variables take the form of Log Y = log a + b × log ESD (cm). AIC = Akaike Information Criterion. * indicates the best model (i.e. lowest value of AIC).

When regressions were significant, slopes and intercepts were compared between raw jellyfish and other pelagic animals and, subsequently between jellyfish standardised for C content and other pelagic animals.

## Results

**Figure 1:**
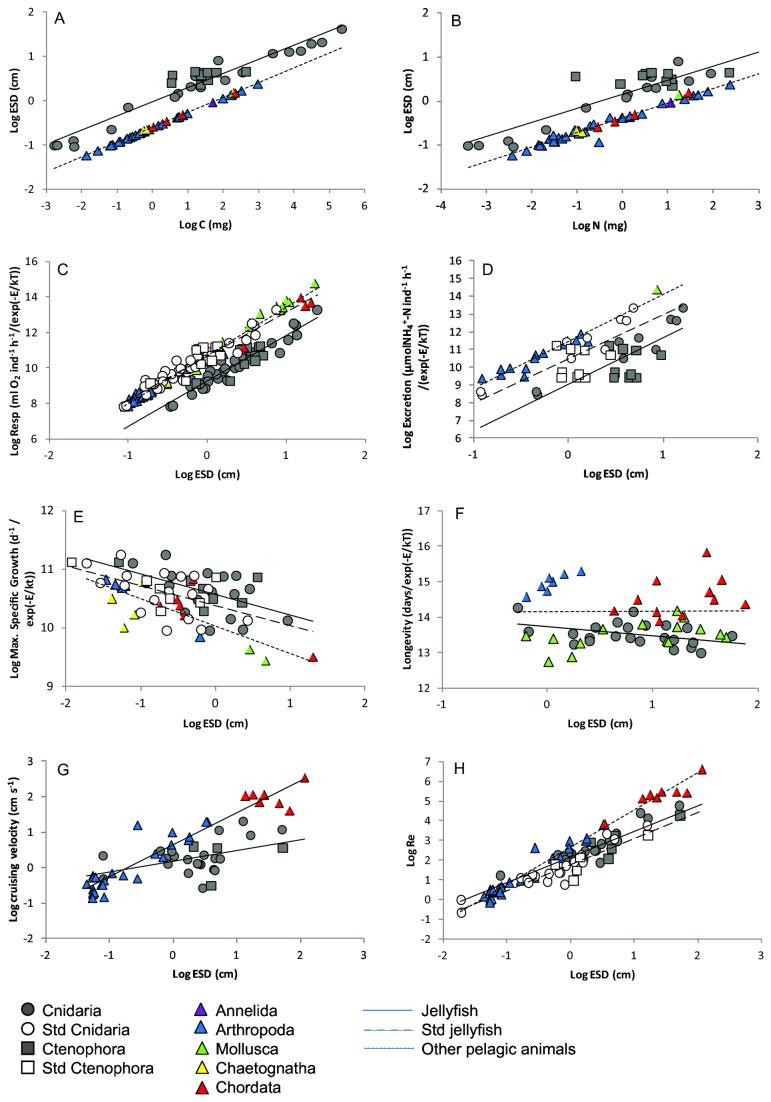
Animal size (equivalent spherical diameter; ESD) as a function of carbon content (A) and nitrogen content (B) for jellyfish and other pelagic animals. Respiration (C), excretion (D), maximum specific growth (E), longevity (F), swimming velocity (G), and Reynolds numbers (H) as a function of ESD for jellyfish, other pelagic animals, and jellyfish whose ESD is standardised for their carbon content. Data and data sources are available in electronic supplementary material (Dataset S1; Appendix S1).

Jellyfish and other pelagic animals displayed similar allometry (i.e. slopes) for carbon and nitrogen content, and rates of respiration, excretion and growth but the intercepts of the relationships for these variables differed. Jellyfish were 3.2 times larger (in terms of ESD) than other pelagic animals of equivalent carbon content and 2.5 times larger than those of equivalent nitrogen content (Table 1, Figure 1A,B). Respiration rates of jellyfish were 28 times slower than those of other pelagic animals of comparable ESD; however, when jellyfish ESD was adjusted for carbon content, the differences in respiration rates were negligible (Table 1, Figure 1C). Excretion rates of jellyfish were 257 times slower than those of other pelagic animals of equivalent ESD. When jellyfish size was adjusted for carbon content, rates of excretion were still 10 times slower than those of other pelagic animals of similar carbon content (Figure 1D). Jellyfish grew 3.5 times faster than other pelagic animals of equivalent ESD and growth rates remained 2.2 times as fast when jellyfish ESD was adjusted for carbon content (Table 1, Figure 1E).

There was a small but significant negative relationship between longevity and size for jellyfish (Table 1, Figure 1F) but when the smallest (which was also the longest lived) medusa, which imposed large leverage on the relationship, was removed medusae exhibited no relationship between longevity and size (*P*=0.273). Medusae generally exhibited seasonal to annual life spans regardless of size and only a few species persisted for up to two years. When considered as a single group, other pelagic animals exhibited no relationship between ESD and longevity (Table 1; Figure 1f) but when analysed separately arthropods (*P*=0.006) and molluscs (*P*=0.047) both exhibited significant positive relationships. When data were corrected for temperature longevity in jellyfish was unrelated to ESD (P=0.331) and other pelagic animals displayed a positive relationship between longevity and ESD (*P*=0.001; Figure S2).

The allometric scaling of swimming velocity differed between jellyfish and other pelagic animals and the slope of the allometric relationship for jellyfish was shallower than that of other pelagic animals (Table 1, Figure 1G). Small jellyfish swam at similar speeds to other pelagic animals of equivalent ESD but large jellyfish swam much more slowly (Figure 1G). Reynolds numbers also scaled allometrically with body size (Table 1, Figure 1H). However, if jellyfish only attained the size equivalent to that reached by other pelagic animals of similar carbon content they would operate in a Re regime approximately one order of magnitude lower than that in which they actually operate. 

## Discussion

The slopes of the allometric relationships for respiration, excretion and specific growth were similar between jellyfish and other pelagic animals but the intercepts differed. Differences in carbon content explained the discrepancy in respiration rates between jellyfish and other pelagic animals but did not fully explain differences in rates of excretion or growth. Indeed even after jellyfish size was adjusted for carbon content, jellyfish excreted nitrogen an order of magnitude more slowly, and grew 3 times faster than other pelagic animals. The allometry of swimming velocity and Re differed between jellyfish and other pelagic animals, indicating fundamental differences in the size-scaling of these properties between groups. Small medusae tended to live slightly longer than large medusae but almost all medusae lived for less than one year, regardless of size. These results suggest that jellyfish are not just low-carbon, high-water analogues of other pelagic body plans, but that they have unique size-dependent traits that cause them to function very differently to other pelagic taxa.

### Jellyfish body plans and consequences for metabolism

The fundamental differences in the body plans of jellyfish and other pelagic taxa appear to have a large influence on metabolic rates. Unlike most metazoans, the bodies of jellyfish comprise thin layers of ectodermal and endodermal tissue that line the external and internal surfaces of their bodies. The bulk of the body consists of the mesoglea, a robust extracellular matrix that comprises water, collagen fibres and salts [20] although in ctenophores, some muscle cells are also located in the mesoglea [21]. The mesoglea provides structural support and has elastic properties that enable it to function as a hydrostatic skeleton, but because it contains few (scyphozoans and ctenophores) or no cells (hydrozoans), its metabolic demand is small [20]. Thus on a wet-weight basis, rates of respiration of jellyfish are much slower than those of other pelagic taxa but when scaled to carbon content, rates of respiration are similar to other metazoans [2].

Despite their similarities in carbon metabolism, jellyfish exhibited substantial differences in nitrogen metabolism, even when their body size was scaled to their carbon content. In the marine environment carbon is usually available in excess but nitrogen is often limiting and this was reflected by jellyfish using nitrogen more efficiently than carbon. The high demand for N by jellyfish reflects the proximate and elemental composition of their body tissues. Jellyfish contain fewer lipids (approximately half those of non-gelatinous groups [22]) and, as a consequence, the ratio of proteins to lipids (~3.3 [13]) is up to twice that of non-gelatinous zooplankton (1.5–2.0 [23]). The relatively high protein and low lipid content is reflected in their molar C:N (4.4 [1]), which is lower than most other zooplankton (4.8-6.2 for crustacean zooplankton [24]) and on par with that of bacteria [25]. A consequence, however, of having C:N lower than their food sources is that jellyfish assimilate more C than required to meet their demand for N. In medusae, excess C is excreted as dissolved organic matter and mucus, leading to large C fluxes from medusae with important implications for carbon budgets and microbial processes [26]. The very high demand for N compared to other metazoans may explain why jellyfish conserve more N compared to other pelagic groups.

### Growth rates

Because the bulk of the bodies of jellyfish are largely acellular and contain high percentages of water, growth rates of jellyfish greatly exceed those of organisms that rely on construction of new tissues to increase in size. Even after adjusting their body size for their low carbon content, jellyfish still grow more than twice as fast as other pelagic taxa of equivalent size suggesting that low carbon content only partly explains their rapid growth rates. Although data are limited, assimilation efficiencies [13] and net growth efficiencies [27,28] of jellyfish are similar to other carnivores [29]. However, their high water content and thus large body size enables them to maintain clearance rates greater than crustacean zooplankton of equivalent carbon content [2]. Consequently jellyfish can acquire more food for a given carbon content than crustacean zooplankton and because they can assimilate it just as efficiently, they can maintain carbon specific growth rates greater than other pelagic animals.

### Swimming and Reynolds numbers

The slope of the allometric relationship for swimming was relatively flat for jellyfish compared to other pelagic taxa; whilst small jellyfish swam at equivalent speeds to other pelagic animals of similar size, large jellyfish swam more slowly. Ctenophores and medusae swim in very different ways. Medusae swim using pulsatile jet propulsion during which contraction of the bell expels a volume of water from behind it, propelling the medusa forward. In small medusae (mostly hydrozoans), this method is highly effective and swimming speeds of up to 13 body lengths s^-1^ can be achieved in short bursts [30]. The streamlined prolate shape of many hydrozoans, coupled with the presence of a skirt-like velum, which narrows the aperture of the contracted bell and thus increases the velocity with which water is expelled, contributes to the effectiveness of this type of locomotion [31]. Larger medusae, however, are oblate rather than prolate-shaped. Jet propulsion is less effective in large oblate individuals because the contractile muscle fibres located in the epithelium of the sub-umbrella are only one cell thick [32] which creates scaling problems as medusae grow larger and the volume of the bell and the force required to contract it increases. Consequently jet propulsion is modified in large oblate medusae to slower “jet paddling” in which a rowing-like movement of the bell margin during the relaxation phase of the swimming cycle produces vortices that counteract the drag-creating vortices generated during the next contraction phase [33]. Although jet-paddling achieves slower speeds for a given size, it is energetically more efficient [34]. Hence although large medusae, such as the scyphozoan *Stomolophus* *meleagris* , swim much more slowly than fish, the efficiency with which they swim is the same [35]. In contrast to medusae, ctenophores cruise slowly by co-ordinated beating of eight rows of ctene plates but lobate forms can also revert to muscular contractions of the lobes to initiate escape responses. Like medusae, small and large ctenophores swim in different ways. The ctenes of small (usually spherical) ctenophores beat metachronously (in sequence) whilst those of large ctenophores beat synchronously [36]. The amount of thrust produced by metachronal beating, however, is constrained by the time it takes the waves to pass over the body of the ctenophore. Hence metachronal beating can only be maintained by small ctenophores and has possibly imposed an evolutionary constraint on the maximum size of ctenophores that use this method [36]. Synchronous beating generates more thrust than metachronal beating which overcomes the increased drag associated with large body size. However, because in large ctenophores each ctene row is innervated independently, more energy may be expended by large ctenophores and, like medusae, larger ctenophores swim relatively more slowly (in terms of body lengths per second) than small ctenophores [36].

The slope of the allometric relationship for Re in jellyfish was shallower than for other pelagic taxa, hence large jellyfish generally experiences a slightly more viscous environment than other pelagic organisms of equivalent size. Most notably, however, jellyfish experienced Re environments that are approximately one order of magnitude greater than if their body sizes were equivalent to that of other organisms with similar carbon content. Hence, having a gelatinous body plan has effectively shifted jellyfish from an intermediate Re region, where both viscous and inertial forces are important, to one where inertial forces dominate. For smaller organisms, viscous effects dominate and boundary layers are thick, thus limiting the delivery of nutrients and other materials to the surface of the organism. The transition from laminar to turbulent flow occurs at Re of ~1000 and, therefore, boundary layers are shed by the majority of jellyfish via swimming or environmental turbulence, which increases contact rates with potential prey. Very small jellyfish (which comprised only hydromedusae in the current study) have smaller body lengths which are expected to lower the Re associated with swimming. However, their prolate body shape and use of jet-propulsion [37] enable small medusae to swim at high instantaneous speeds, and equivalent or even higher Re compared with other plankton of similar size. Therefore, being gelatinous helps to free jellyfish from some of the hydrodynamic constraints associated with small body size.

Although the effects of inertia dominate during swimming, jellyfish (except for lobate ctenophores) capture prey using tentacles, which have much smaller length scales (diameter ~0.01-0.1 mm [38]). Therefore, if we assume a velocity of 1 cm s^-1^ at the scale of the tentacles, Re are in an intermediate range (0.1-1), where viscous and inertial forces are both important. Within this intermediate range, boundary layers around the feeding elements are more compressed than they would be at lower Re (<<0.1) resulting in higher particle capture rates [39] compared to feeding structures of plankton that operate at low Re. A range of taxa take advantage of feeding at intermediate Re so this aspect may not be unique to jellyfish [39]. However, jellyfish display a unique combination of swimming and predatory efficiency [2] that may contribute to their overall evolutionary success.

### Longevity

Most medusae live for less than one year and when *Aglanthadigitale* (1 of 23 data points for medusae that created large leverage on the analysis) was omitted, longevity in medusae was independent of size. Independence between longevity and size in medusae is highly unusual because allometric scaling between longevity and body size has been recognized for more than a century and appeared to conform across protists, plants, and animals [40]. However, when corrected for temperature, longevity in other pelagic taxa also initially appeared independent of size but this was driven by large differences in the allometric scaling between the arthropods (euphausiids), molluscs (cephalopods) and chordates (fish) that were included in the analysis. Indeed, when each phylum was analysed separately, positive allometric relationships were revealed for arthropods and molluscs and a general positive increase in longevity with body size was observed for all other pelagic taxa when data were not corrected for temperature. Hence the lack of size-scaling in medusae is unusual. Increased production of reactive oxygen species (the so-called oxidative stress hypothesis) via peroxidation of fatty acids in cellular membranes, and differences in the fatty acid composition of animals of different sizes, may provide the mechanistic basis for the scaling between longevity and size [40]. The lack of size-scaling with longevity in medusae could reflect similarities in the fatty acid composition of membranes across taxa of various sizes but fatty acid compositions of too few species of medusae are available to test this hypothesis. Moreover, a lack of investment in antioxidant defences with increasing body size might likewise result in a constant rate of senescence across size classes of jellyfish [41].

Longevity may be governed by extrinsic (e.g. environmental conditions, predation, disease, parasitism and food limitation) as well as intrinsic factors [42]. The short lifespan of medusae in the field, and observations that medusae can survive for more than two years in captivity [18] may indicate that extrinsic factors govern longevity in wild populations. Indeed, medusae frequently exhibit increased physical damage and parasitic loads prior to the annual disappearance of populations [43,44]. Some species tolerate much narrower fluctuations in temperature and salinity than their habitat experiences throughout the year, and longevity in these species is thus tightly coupled to seasonal changes in physical conditions [45]. For other species, ecological interactions may determine longevity. Indeed, the ability of medusae to proliferate and grow rapidly means that competition for food is intense. The large size and high clearance rates of medusae in combination with their low carbon content theoretically enables medusae to survive at lower prey concentrations than other zooplankton [2] but dense populations of medusae are usually only sustained when productivity is high (e.g. [46]), presumably because growth rates are rapid. The paucity of lipid reserves in the bodies of medusae means that individuals quickly shrink when deprived of food [47]. Although medusae may sustain prolonged periods of starvation in the laboratory [47] examples of cohorts of medusae shrinking in the field are rare and usually precede the disappearance of a population (e.g. [44]) suggesting that poor body condition associated with starvation may render individuals more susceptible to disease or predation. Consequently, despite the large size of some medusae, longevity is generally short and often correlated with seasonal cycles in productivity and reproduction.

The bipartite life history of scyphozoan and many hydrozoan medusae may also reduce the need for the sexually-reproducing medusae to be long-lived. This is because the major role of the sexually reproducing medusae may be to maintain genetic diversity rather than to facilitate repopulation. Indeed the annual populations of medusae are restocked from asexual reproduction of benthic polyps. In some scyphozoans up to 40 medusae may bud from a single polyp at a time and some polyps can bud repeatedly (e.g. [48]). Prolific asexual reproduction of the polyps means that a single sexually-produced larva could, theoretically, give rise to almost infinite numbers of medusae. Thus the major role of the medusae may be to simply maintain genetic diversity whilst the annual restocking of the population may be facilitated by the asexual polyps.

### Jellyfish differ in more than just low carbon content

The high water and low carbon content contributes to the evolutionary longevity and widespread distribution and abundance of jellyfish [2,49]; but alone, these factors do not suffice to explain the multiple anomalies in the allometric relationships presented by jellyfish. Indeed the most outstanding anomalies of jellyfish are their difference in scaling (i.e. slope) for swimming speed with size and the relatively short, seasonal life spans exhibited by animals that can weigh up to 200kg. The key to the short life-span of medusae may be their low construction costs, because the construction costs of a carbon-based organism of comparable size would make it very inefficient to have short life-spans. However, because construction costs of medusae are small due to their high water content, they can afford the anomalous combination of large size and short life-span. Consequently emergent properties of jellyfish associated with their size and evolved life histories are required to more completely explain why jellyfish body plans have been conserved for half a billion years, as well as to predict their shifting role in a changing ocean.

Jellyfish are renowned for forming spectacular population blooms in coastal waters throughout the world. Metabolic rate determines the amount of resources required to sustain a given biomass [50]. Because metabolic rates of jellyfish are 1-2 orders of magnitude slower than non-gelatinous organisms of equivalent ESD, a given amount of resources could sustain a much greater wet biomass of medusae than non-gelatinous organisms. Thus medusae have a greater propensity to form blooms than non-gelatinous organisms. Moreover, slow rates of respiration, coupled with the ability to store oxygen within the mesoglea [51], may predispose medusae for survival in low oxygen environments. Because hypoxia is spreading rapidly in the world’s oceans [52], the area habitable by jellyfish, to the exclusion of other taxa, may likewise be increasing.

## Conclusions

The watery body plans of jellyfish appear to confer multiple adaptive advantages but several of the ecological advantages cannot be solely explained by their high water and low carbon content. Rather, it is emergent properties associated with their large size, simple body plan, and complex life histories that most likely explains the capacity of jellyfish to proliferate and develop spectacular blooms and may enable these animals to impact their environment in ways that are disproportionate to their size or carbon content.

## Supporting Information

Figure S1 **Rates of respiration as a function of ESD for jellyfish and other epipelagic and meso-bathypelagic pelagic taxa.** Data sources are available in Dataset S2.
(EPS)

Figure S2 **Longevity as a function of ESD for jellyfish and other pelagic taxa uncorrected for temperature**. Data sources are available in Dataset S1.
(EPS)

Dataset S1 **Complete set of data used in analyses.**
(XLSX)

Dataset S2 **Data used to compare rates of respiration of jellyfish and other pelagic taxa from different depths.**
(XLSX)

Appendix S1 **Reference list for data used in Dataset S1.**
(DOCX)
