# Supplementary material for: Jellyfish Body Plans Provide Allometric Advantages beyond Low Carbon Content
Source: PLoS One. 2013 Aug 13;8(8):e72683. doi: 10.1371/journal.pone.0072683 (PMC3742524; doi:10.1371/journal.pone.0072683)
Supplement: Appendix S1 — Reference list for data used in Dataset S1. (DOCX) [file pone.0072683.s005.docx]

**Appendix S1. References for data used in analyses**

1. Bailey TG, Youngbluth MJ, Owen GP (1995) Chemical composition and metabolic rates of gelatinous zooplankton from midwater and benthic boundary layer environments off Cape Hatteras, North Carolina, USA. Mar Ecol Prog Ser 122: 121-134.
2. Matsakis S, Conover RJ (1991) Abundance and feeding of medusae and their potential impact as predators on other zooplankton in Bedford Basin (Nova Scotia, Canada) during spring. Can J Fish Aquat Sci 48: 1419-1430.
3. Clarke A, Holmes LJ, Gore DJ (1992) Proximate and elemental composition of gelatinous zooplankton from the Southern Ocean. J Exp Mar Biol Ecol 155: 5-68.
4. Matsakis S, Conover RJ (1991) Abundance and feeding of medusae and their potential impact as predators on other zooplankton in Bedford Basin (Nova Scotia, Canada) during Spring. Can J Fish Aquat Sci 48: 1419-1430.
5. Matsakis S, Nival P (1989) Elemental composition and food intake of *Phialidium* hydromedusae in the laboratory. J Exp Mar Biol Ecol 130: 277-290.
6. Shenker JM (1985) Carbon content of the neritic scyphomedusa *Chrysaora fuscescens*. J Plankton Res 7: 169-173.
7. Youngbluth MJ, Båmstedt U (2001) Distribution, abundance, behaviour and metabolism of *Periphylla periphylla*, a mesopelagic coronate medusa in a Norwegian fjord. Hydrobiologia 451: 321-333.
8. Finenko GA, Romanova ZA, Abolmasova GI, Anninsky BE, Svetlichny LS, Hubareva ES, Bat L, Kideys AE (2003) Population dynamics, ingestion, growth and reproduction rates of the invader *Beroe ovate* and it impact on plankton community in Sebastopol Bay, the Black Sea. J Plankton Res 25: 539-549.
9. Ikeda T, Bruce B (1986) Metabolic activity and elemental composition of krill and other zooplankton from Prydz Bay, Antarctica, during early summer (November-December). Mar Biol 92: 545-555.
10. Ikeda T, Mitchell AW (1982) Oxygen uptake, ammonia excretion and phosphate excretion by krill and other Antarctic zooplankton in relation to their body size and chemical composition. Mar Biol 71: 283-298.
11. Kremer P (1975) Excretion and body composition of the ctenophore *Mnemiopsis leidyi* (A. Agassiz): comparisons and consequences. 10^th^ European Symposium on Marine Biology. 2: 351-362.
12. Reeve MR, Baker LD (1975) Production of two planktonic carnivores (chaetognath and ctenophore) in South Florida inshore waters. Fish Bull 73: 238-248.
13. Kremer P, Canino MF, Gilmer RW (1986) Metabolism of epipelagic tropical ctenophores. Mar Biol 90: 403-412.
14. Omori M (1969) Weight and chemical composition of some important oceanic zooplankton in the North Pacific Ocean. Mar Biol 3: 4-10.
15. Larson RJ (1987) Costs of transport for the scyphomedusa *Stomolophus meleagris* L. Agassiz. Can J Zool 65: 2690-2695.
16. Ikeda T, Skjoldal HR (1989) Metabolism and elemental composition of zooplankton from the Barents Sea during early Arctic summer. Mar Biol 100: 173-183.
17. Thuesen EV, Childress JJ (1994) Oxygen consumption rates and metabolic enzyme activities of oceanic California medusae in relation to body size and habitat depth. Biol Bull 187: 84-98.
18. Marshalonis D, Pickney JL (2007) Respiration rates in dominant hydromedusae in the North inlet tidal estuary during winter and summer. J Plankton Res 29: 1031-1040.
19. Kinoshita J, Hiromi J, Kadota S (1997). Do respiratory metabolic rates of the scyphomedusa *Aurelia aurita* scale isometrically throughout ontogeny in a sexual generation? Hydrobiologia 347: 51-55.
20. Uye S-I, Shimauchi H (2005) Population biomass, feeding, respiration and growth rates, and carbon budget of the scyphomedusa *Aurelia aurita* in the Inland Sea of Japan. J Plankton Res 27: 237-248.
21. Ishii H, Tanaka F (2006) Respiration rates and metabolic demands of *Aurelia aurita* in Tokyo Bay with special reference to large medusae. Plankton and Benthos Research 1: 64-67.
22. Purcell JE, Fuentes V, Atienza D, Tilves U, Astorga D, et al. (2010) Use of respiration rates of scyphozoan jellyfish to estimate their effects on the food web. Hydrobiologia 645: 135-152.
23. Davenport J, Trueman ER (1985) Oxygen uptake and buoyancy in zooplanktonic organisms from the tropical eastern Atlantic. Comp Biochem Physiol 81A: 857-863.
24. Kideys AE, Finenko GA, Anninsky BE, Shiganova TA, Roohi A, Tabari MR, Youseffyan M, Rostamian MT, Rostami H, Negarestan H (2004) Physiological characteristics of the ctenophore *Beroe ovate* in Caspian Sea water. Mar Ecol Prog Ser 266:111-121.
25. Svetlichny LS, Abolmasova GI, Hubareva ES, Finenko GA, Bat L, Kideys AE (2004) Respiration rates of *Beroe ovata* in the Black Sea. Mar Biol 145: 585-593.
26. Møller LF, Canon JM, Tiselius P (2010) Bioenergetics and growth in the ctenophore *Pleurobrachia pileus*. Hydrobiologia 645: 167-178.
27. Ikeda T, Kanno Y, Ozaki K, Shinada A (2001) Metabolic rates of epipelagic marine copepods as a function of body mass and temperature. Mar Biol 139: 587-596.
28. Seibel BA, Drazen JC (2007) The rate of metabolism in marine animals: environmental constraints, ecological demands and energetic opportunities. Phil Trans R Soc B 362: 2061-2078.
29. Webber DM, O’Dor RK (1985) Respiration and swimming performance of short-finned squid (*Illex illecebrosus*). NAFO Sci Coun Studies 9: 133-138.
30. Ikeda T (1996) Metabolism, body composition and energy budget of the mesopelagic fish *Maurolicus muelleri* in the Sea of Japan. Fish Bull 94: 49-58.
31. Steinhausen MF, Steffensen JF, Andersen NG (2005) Tail beat frequency as a predictor of swimming speed and oxygen consumption of saithe (*Pollachius virens*) and whiting (*Merlangius merlangus*) during forced swimming. Mar Biol. 148: 197-204.
32. Matsakis S (1992) Ammonia excretion rate of *Clytia* spp. Hydromedusae (Cnidaria, Thecata): effects of individual dry weight, temperature and food availability. Mar Ecol Prog Ser 87: 55-63.
33. Ikeda T, Fay EH, Hutchison SA, Boto GM (1982). Ammonia and inorganic phosphate excretion by zooplankton from inshore waters of the Great Barrier Reef, Queensland. I Relationships between excretion rates and body size. Aust J Mar Freshw Res 33: 55-70.
34. Shimauchi H, Uye S-I (2007) Excretion and respiration rates of the scyphomedusa *Aurelia aurita* from the Inland Sea of Japan. J Oceanogr 63: 27-34.
35. Condon RH, Steinberg DK, Bronk DA (2010). Production of dissolved organic matter and inorganic nutrients by gelatinous zooplankton in the York River estuary, Chesapeake Bay. J Plankton Res 32: 153-170.
36. Nemazie DA, Purcell JE, Gilbert PM (1993) Ammonium excretion by gelatinous zooplankton and their contribution to the ammonium requirements of microplankton in Chesapeake Bay. Mar Biol 116: 451-458.
37. Pitt KA, Koop K, Rissik D ( 2005) Contrasting contributions to inorganic nutrient recycling by the co-occuring jellyfishes, *Catostylus mosaicus* and *Phyllorhiza punctata* (Scyphozoa, Rhizostomeae). J Exp Mar Biol Ecol 315: 71-86.
38. Morand P, Carré C, Biggs D (1987) Feeding and metabolism of the jellyfish *Pelagia noctiluca* (Scyphomedusae, Semaeostomeae). J Plankton Res 9: 651-665.
39. Malej A (1989) Respiration and excretion rates of *Pelagia noctiluca* (Semaeostomeae, Scyphozoa). Proceedings of the 21^st^ EMBS pp 107-113.
40. Youngbluth MJ, Kremer P, Bailey TG, Jacoby CA (1988) Chemical composition, metabolic rates and feeding behaviour of the midwater ctenophore *Bathocyroe fosteri*. Mar Biol 98: 87-94.
41. Kremer P (1982) Effect of food availability on the metabolism of the ctenophore *Mnemiopsis mccradyi*. Mar Biol 71: 149-156.
42. Yamada Y, Ikeda T (2003) Metabolism and chemical composition of four pelagic amphipods in the Oyashio region, western subarctic Pacific Ocean. Mar Ecol Prog Ser 253: 233-241.
43. Boucher-Rodoni R, Mangold K (1989) Respiration and nitrogen excretion by the squid *Loligo forbesi*. Mar Biol 103 333-338.
44. Hirst AG, Roff JC, Lampitt RS (2003) A synthesis of growth rates in marine epipelagic invertebrate zooplankton. Adv Mar Biol 44: 1-141.
45. Møller LF, Riisgård HU (2007) Population dynamics, growth and predation impact of the common jellyfish *Aurelia aurita* and two hydromedusae, *Sarsia tubulosa*, and *Aequorea vitrina* in Limfjorden (Denmark). Mar Ecol Prog Ser 346: 153-165.
46. Purcell JE, Båmstedt U, Båmstedt A (1999) Prey, feeding rates, and asexual reproduction rates of the introduced oligohaline hydrozoans *moerisia lyonsi*. Mar Biol 134: 317-325.
47. Olesen NJ, Frandsen K, Riisgård HU (1994) Population dynamics, growth and energetic of jellyfish *Aurelia aurita* in a shallow fjord. Mar Ecol Prog Ser 105: 9-18.
48. Miyake H, Iwao K, Kakinuma Y (1997) Life history and environment of *Aurelia aurita*. South Pacific Study 17 273-285.
49. Hernroth L,Gröndahl F (1983). On the biology of *Aurelia aurita* (L.) 1. Release and growth of *Aurelia aurita* (L.) ephyrae in the Gullmar Fjord, Western Sweden, 1982-83. Ophelia 22 189-199.
50. Yasuda T (1971) Ecological studies on the jelly-fish, *Aurelia aurita* in Urzoko Bay, Fukui Prefecture – IV. Monthly change in the bell-length composition and breeding season. Bull Jap Soc Sci Fish 37: 364-370.
51. Hamner WM, Jenssen RM (1974) Growth, degrowth, and irreversible cell differentiation in *Aurelia aurita*. Am Zool 14: 833-849.
52. Möller H (1980) Population dynamics of *Aurelia aurita* medusae in Kiel Bight, Germany (FRG). Mar Biol 60: 123-128.
53. Pitt KA, Kingsford MJ (2003) Temporal and spatial variation in recruitment and growth of medusae of the jellyfish, *Catostylus mosaicus* (Scyphozoa: Rhizostomeae). Mar Freshw Res 51: 117-125.
54. Kikinger R (1992) *Cotylorhiza tuberculata* (Cnidaria: Scyphozoa) – life history of a stationary population. Mar Ecol 13: 333-362.
55. Brewer RH (1989) The annual pattern of feeding, growth, and sexual reproduction in *Cyanea* (Cnidaria: Scyphozoa) in the Niantic River Estuary, Connecticut. Biol Bull 176: 272-281.
56. Kawahara M, Uye S-I, Ohtsu K, Iizumi H (2006) Unusual population explosion of the giant jellyfish, *Nemopilema nomurai*, (Scyphozoa: Rhizostomeae) in east Asian waters. Mar Ecol Prog Ser 307: 161-173.
57. Grove M, Breitburg DL (2005) Growth and reproduction of gelatinous zooplankton exposed to low dissolved oxygen. Mar Ecol Prog Ser 301: 185-198.
58. Hanlon TR, Hixon RF, Hulet WH (1983) Survival, growth and behaviour of the loliginid squids *Loligo plei*, *Loligo pealei*, and *Lolliguncula brevis* (Mollusca: Cephalopoda) in closed seawater systems. Biol Bull 165: 63.7-685.
59. Hunter JR (1976) Culture and growth of northern anchovy, *Engraulis mordax*, larvae. Fish Bull 74: 81-88.
60. Frommel A, Clemmesen C (2009) Use of biochemical indices for analysis of growth in juvenile two-spotted gobies (*Gobiusculus flavescens*) of the Baltic Sea. Sci Mar 73S1: 159-170.
61. Simms JR, Rooker JR, Holt SA, Holt GJ, Bangma J (2010) Distribution, growth, and mortality of sailfish (*Istiophorus* platypterus) larvae in the northern Gulf of Mexico. Fish Bull 108: 478-190.
62. Faulk CK, Holt GJ (2005) Advances in rearing cobia *Rachycentron canadum* larvae in recirculating aquaculture systems: Life prey enrichment and greenwater culture. Aquaculture 249: 231-243.
63. Hunter JR, Kimbrell CA (1980) Early life history of Pacific mackerel, *Scomber japonicus.* Fishery Bulletin 78: 89-101.
64. Edwards RRC, Finlayson DM, Steele JH (1972) An experimental study of the oxygen consumption, growth, and metabolism of the cod (*Gadus morhua* L.). J Exp Mar Biol Ecol 8: 299-309.
65. Edwards C (1973) The medusa *Mitrocomella polydiademata* and its hydroid. J Mar Biol Ass UK 53: 601-607.
66. Mills CE (1993) Natural mortality in NE Pacific coastal hydromedusae: grazing predation, wound healing and senescence. Bull Mar Sci 53: 194-203.
67. Lucas CH, Williams DW, Williams JA, Sheader M (1995) Seasonal dynamics and production of the hydromedusan *Clytia hemisphaerica* (Hydromedusa: Leptomedusa) in Southampton Water. Estuaries 18: 362-372.
68. Pertsova NM, Kosobokova KN, Prudkovsky AA (2006) Population size structure, spatial distribution, and life cycle of the Hydromedusa *Aglantha digitale* (O.F. Müller, 1766) in the White Sea. Oceanology 46: 249-258.
69. Lucas CH, Hirst AG, Williams JA (1997) Plankton dynamics and *Aurelia aurita* production in two contrasting ecosystems: comparisons and consequences. Est Coast Shelf Sci 45: 209-219.
70. Lucas CH, Williams JA (1994). Population dynamics of the Scyphomedusa *Aurelia aurita* in Southampton Water. J Plankton Res 16: 879-895.
71. Schneider G, Behrends G (1994) Population dynamics and the trophic role of *Aurelia aurita*  medusae in the Kiel Bight and western Baltic. ICES J Mar Sci 51: 359-367.
72. Decker MB, Brown CW, Hood RR, Purcell JE, Gross TF, et al. (2007) Predicting the distribution of the scyphomedusa *Chrysaora quinquecirrha* in Chesapeake Bay. Mar Ecol Prog Ser 329: 99-113.
73. Uye S-I (2008) Blooms of the giant jellyfish *Nemopilema nomurai*: a threat to fisheries sustainability of the East Asian Marginal Seas. Plankton Benth Res 3: 125-131.
74. Malej A, Malej M (1992) Population dynamics of the jellyfish *Pelagia noctiluca* (Forsskål, 1775). Proceedings of the 25^th^ EMBS pp 215-219.
75. Haddad MA, Nogueira M (2006) Reappearance and seasonality of *Phyllorhiza punctata* von Lendenfeld (Cnidara, Scyphozoa, Rhizostomeae) medusae in southern Brazil. Revista Brasileira de Zootecnia, 23: 824-831.
76. Rippingale RJ, Kelly SJ (1995) Reproduction and survival of *Phyllorhiza punctata* (Cnidaria: Rhizostomeae) in a seasonally fluctuating salinity regime in Western Australia. Mar Freshw Res, 46: 1145-1151.
77. Graham WM, Martin DL, Felder DL, Asper VL, Perry HM (2003) Ecological and economic implications of a tropical jellyfish invader in the Gulf of Mexico. Biol Invasions 5: 53-69.
78. Taki K, Yabuki T, Noiri Y, Hayashi T, Naganobu M (2008) Horizontal and vertical distribution and demography of euphausiids in the Ross Sea and its adjacent waters in 2004/2005. Polar Biol 31: 1343-1356.
79. Astthorsson OS, Gislason A (1997) Biology of euphausiids in the subarctic waters north of Iceland. Mar Biol 129 319-330.
80. Dalpadado P, Skjoldal HR (1996) Abundance, maturity and growth of the krill species *Thysanoessa inermis* and *T. longicaudata* in the Barents Sea. Mar Ecol Prog Ser 144: 175-183.
81. Young RE, Mangold KM (1994) Growth and reproduction in the mesopelagic-boundary squid *Abralia trigonura*. Mar Biol 119: 413-421.
82. Markaida U, Quiñónez-Velázquez C, Sosa-Nishizaki O (2004) Age, growth and maturation of jumbo squid *Dosidicus gigas* (Cephalopoda: Ommastrephidae) from the Gulf of California, Mexico. Fish Res 66: 31-47.
83. Tracey SR, Steer MA, Pecl GT (2003) Life history traits of the temperate mini-maximalist *Idiosepius notoides*, (Cephalopoda: Sepioidea). J Mar Biol Ass UK 83: 1297-1300.
84. Sato N, Kasugai T, Munehara H (2008) Estimated life span of the Japanese pygmy squid, *Idiosepius paraodxus* from statolith growth increments. J Mar Biol Ass UK 88: 391-394.
85. Jackson GD (1989) The use of statolith microstructures to analyse life-history events in the small tropical cephalopod *Idiosepius pygmaeus*. Fish Bull 87: 265-272*.*
86. Arkhipkin A (1993) Age, growth, stock structure and migratory rate of pre-spawning shot-finned squid *llex argentines* based on statolith ageing investigations. Fish Res 16: 313-338.
87. Arkhipkin A, Jereb P, Ragonese S (2000) Growth and maturation in two successive seasonsl groups of the short-finned squid, *Illex coindetii* from the Strait of Sicily (central Mediterranean). ICES J Mar Sci 57: 31-41.
88. Hatfield EMC (2000) Do some like hot? Temperature as a possible determinant of variability in the growth of the Patagonian squid, *Loligo gahi* (Cephalopoda: Loliginidae). Fish Res 47: 27-40.
89. Butler J, Fuller D, Yaremko M (1999) Age and growth of market squid (*Loligo opalescens*) off California during 1998. CalCOFI Report 40: 191-195.
90. Brodziak JKT, Macy WK (1996) Growth of long-finned squid, *Loligo pealei*, in the northwest Atlantic. Fish Bull 94: 212-236.
91. Arkhipkin A. (1995) Age, growth and maturation of the European squid *Loligo vulgaris* (Myopsida, Loliginidae) on the West Saharan Shelf. J Mar Biol Ass UK 75: 593-604.
92. Dimmlich WF, Hoedt FE (1998) Age and growth of the myosid squid *Loliolus noctiluca* in Western Port, Victoria, determined from statolith microstructure analysis. J Mar Biol Ass UK 78: 277-586.
93. Wang K-Y, Lee K-T, Liao C-H (2010) Age, growth and maturation of swordtip squid (*Photololigo edulis*) in the southern east China Sea. J Mar Sci Tech 18: 99-105.
94. Arkhipkin AI (1997) Age of the micronektonic squid *Pterygioteuthis gemmata* (Cephalopoda: Pyroteuthidae) from the central-east Atlantic based on statolith growth increments. J Moll Stud 63: 287-290.
95. Nigmatullin CM, Arkhipkin AI, Sabirov RM (1995) Age, growth and reproductive biology of diamond-shaped squid *Thysanoteuthis rhombus* (Oegopsida: Thysanoteuthidae). Mar Ecol Prog Ser 124: 73-87.
96. Froese R, Pauly D. Editors (2012) FishBase. World Wide Web electronic publication. [www.fishbase.org](http://www.fishbase.org), version (12/2012).
97. Shorten M, Davenport J, Seymour JE, Cross MC, Carrette TJ, et al. (2005) Kinematic analysis of swimming in Australian box jellyfish, *Chiropsalmus* sp. and *Chironex fleckeri* (Cubozoa, Cnidaria: Chirodropidae). J Zool 267: 371-380.
98. Larson RJ, Mills CE, Harbison GR (1989) *In situ* foraging and feeding behaviour of narcomedusae (Cnidaria: Hydrozoa). J Mar Biol Ass UK 69: 785-794.
99. Colin SP, Costello JH (1996) Relationship between morphology and hydrodynamics during swimming by the hydromedusae *Aequorea victoria* and *Aglantha digitale*. Sci Mar 60: 35-42.
100. Gladfelter WG (1973) A comparative analysis of the locomotory systems of medusoid Cnidaria. Helgoländer wiss. Meeresunters 25: 228-272.
101. Mills CE (1981) Diversity of swimming behaviours in hydromedusae as related to feeding and utilization of space. Mar Biol 64: 185-189.
102. McHenry MJ, Jed J (2003) The ontogenetic scaling of hydrodynamics and swimming performance in jellyfish *(Aurelia aurita*). J Exp Biol 206: 4125-4137.
103. Costello JH, Colin SP (1994) Morphology, fluid motion and predation by the scyphomedusa *Aurelia aurita.* Mar Biol 121: 327-334.
104. Pitt KA, Kingsford MJ (2000) Geographic separation of stocks of the edible jellyfish *Catostylus mosaicus* (Rhizostomeae) in New South Wales, Australia. Mar Ecol Prog Ser 196:143-155.
105. Mataoniski JC, Hood RR, Purcell JE (2001) Characterizing the effect of prey on swimming and feeding efficiency of the scyphomedusa *Chrysaora quinquecirrha*. Mar Biol 139 191-200.
106. Ford MD, Costello JH, Heidelberg KB, Purcell JE (1997) Swimming and feeding by the scyphomedusa *Chrysaora quinquecirrha*. Mar Biol 129: 355-362.
107. Costello JH, Colin SP (1995) Flow and feeding by swimming scyphomedusae. Mar Biol 124: 399-406.
108. Higgins JE, Ford MD, Costello JH (2008) Transition in morphology, nematocyst distribution, fluid motions, and prey capture during development of the scyphomedusa *Cyanea capillata*. Biol Bull 214: 29-41.
109. Honda N, Matsushita Y (2009) *In situ* measurement of swimming speed of giant jellyfish *Nemopilema nomurai*. Nippon Suisan Gakkaishi 75: 701-703.
110. D’Ambra Costello JH, Bentivegna F (2001) Flow and prey capture by the scyphomedusa *Phyllorhiza punctata* von Lendenfeld, 1884. Hydrobiologia 451: 223-227.
111. Matsumoto GI (1991) Swimming movements of ctenophores, and the mechanics of propulsion by ctene rows. Hydrobiologia 216/217: 319-325.
112. Matsumoto GI, Harbison GR (1993) In situ observations of foraging, feeding, and escape behaviour in three orders of oceanic ctenophores: Lobata, Cestida, and Beroida. Mar Biol 117: 279-287.
113. Kreps TA, Purcell JE, Heidelberg KB (1997) Escape of the ctenophore *Mnemiopsis leidyi* from the scyphomedusa predator *Chrysaora quinquecirrha*. Mar Biol 128: 441-446.
114. Huntley ME, Zhou M (2004) Influence of animals on turbulence in the sea. Mar Ecol Prog Ser 273: 65-79.
115. Kiørboe T (2008) Optimal swimming strategies in mate-searching pelagic copepods. Oecologia 155: 179-192.
116. Yen J, Rasberry KD, Webster DR (2008) Quantifying copepod kinematics in a laboratory turbulence apparatus. J Mar Sys 69: 283-294.
117. Price HJ (1989) Swimming behaviour of krill in response to algal patches: A mesocosm study. Limnol Oceanogr 34: 649-659.
118. He P, Wardle CS (1988) Endurance at intermediate swimming speeds of Atlantic mackerel, *Scomber scombrus* L., herring, *Clupea harengus* L., and saithe, *Pollarchius virens* L. J Fish Biol 33: 255-266.
119. Breen M, Dyson J, O’Neill FG, Jones E,Haigh M (2004) Swimming endurance of haddock (*Melanogrammus* aeglefinus L.) at prolonged and sustained swimming speeds, and its role in their capture by towed fishing gears. ICES J Mar Sci 61: 1071-1079.
120. Beamish FWH (1984) Swimming performance of three southwest Pacific Fishes. Mar Biol 79: 311-313.
121. Thuesen EV, Childress JJ (1993) Metabolic rates, enzyme activities and chemical compositions of some deep=sea pelagic worms, particularly *Nectonemertes mirabilis* (Nemertea; Hoplonemertinea) and *Poeobius meseres* (Annelida; Polychaeta). Deep-Sea Res 40: 937-951.
122. Cowles DL, Childress JJ, Wells ME (1991) Metabolic rates of midwater crustaceans as a function of depth of occurrence off the Hawaiian Islands: food availability as a selective factor? Mar Biol 110: 75-83.
123. Seibel BA, Thuesen EV, Childress JJ, Gorodezky LA (1997) Decline in pelagic cephalopod metabolism with habitat depth reflects differences in locomotory efficiency. Biol Bull 192: 262-278.
